# The Epidemiology of Appendicitis and Appendectomy in South Korea: National Registry Data

**DOI:** 10.2188/jea.JE20090011

**Published:** 2010-03-05

**Authors:** Jung Hun Lee, Young Sun Park, Joong Sub Choi

**Affiliations:** 1 Department of Obstetrics and Gynecology, Kangbuk Samsung Hospital, Sungkyunkwan University School of Medicine, Seoul, Korea; 2 Department of Mathematics, College of Natural Sciences, Hanyang University, Seoul, Korea

**Keywords:** appendectomy, appendicitis, epidemiology, health insurance claims

## Abstract

**Background:**

Appendicitis is one of the most frequent acute surgical conditions of the abdomen, and appendectomy is one of the most commonly performed operations in the world. However, epidemiological data on appendicitis have not been reported for South Korean or East Asian populations.

**Methods:**

We analyzed the epidemiological features and lifetime risk of appendicitis and appendectomy in South Korea using data collected for the national health insurance database from 2005 through 2007.

**Results:**

Appendectomy was performed in 59.70% of inpatients diagnosed with appendicitis. The overall incidences of appendicitis, total appendectomy, and perforated appendectomy were 22.71, 13.56, and 2.91 per 10 000 population per year, respectively. The incidence of appendicitis and appendectomy showed clear seasonality, with a peak in summer. The standardized lifetime risks of appendicitis and appendectomy were constant from 2005 through 2007. A life table model suggests that the lifetime risk of appendicitis is 16.33% for males and 16.34% for females, and that the lifetime risk of appendectomy is 9.89% for males and 9.61% for females.

**Conclusions:**

As compared to results obtained in research on Western populations, appendicitis and appendectomy had a similar perforation rate and seasonality, but a higher overall incidence, in South Koreans. Between 2005 and 2007, the incidence of appendicitis and appendectomy was constant. Overall, an estimated 15 incidental appendectomies are performed to prevent 1 inpatient with suspected appendicitis, and 26 incidental appendectomies are performed to prevent 1 appendectomy. Incidental appendectomy may have greater preventive value in Koreans.

## INTRODUCTION

Appendicitis is one of most common acute surgical conditions of the abdomen and appendectomy is one of the most frequently performed operations in the world.^1^ Although numerous reports have been published on the epidemiology of appendicitis, most describe Western populations.^2^^–^^8^ However, because of differences in race, geography, climate, and dietary intake of fiber,^2^ the epidemiological features of appendicitis in South Korea may differ from those of Western populations. To our knowledge, no epidemiological data on appendicitis in South Korean or other Asian populations have been published. In addition, although incidental appendectomy is commonly performed during other abdominal or pelvic surgery, there are no studies of the lifetime risk of appendicitis in Asia. Hence, the preventive value of incidental appendectomy performed in Asia has not been evaluated.

The purpose of this study was to investigate the epidemiological features and lifetime risk of appendicitis and appendectomy in South Korea using data from the national health insurance database that were collected from 2005 through 2007.

## METHODS

### Data source

We obtained the national health insurance claims data collected by the Health Insurance Review Agency (HIRA) of South Korea. The HIRA is a governmental agency overseen by the Korean Ministry of Health and Welfare, and it examines and evaluates the medical expenses of all citizens (approximately 49 million people) covered by the Korean National Health Insurance (approximately 96.6% of the population) and Medical Aid (approximately 3.4%). The HIRA database contains reimbursement records from all medical facilities in South Korea (5–6 million inpatient visits per year at approximately 1100 hospitals and 25 000 private clinics).^9^

Thirteen diagnoses, according to the ICD-10, and 2 surgical procedures, according to the Health Insurance Reimbursement guide issued by the Korean Ministry of Health and Welfare, are coded as appendiceal disease and appendectomy, respectively, in the HIRA data set. The 2 surgical procedure codes were Q2861 (Appendectomy for non-perforated appendicitis) and Q2862 (Appendectomy for perforated appendicitis). We used the HIRA data set to collect information on the number, age group, sex, month, and discharge diagnosis of inpatients who had (1) appendiceal disease (diagnostic code K35–K38.9) as the primary discharge diagnosis or (2) appendiceal disease as the primary discharge diagnosis and had undergone appendectomy (procedure codes Q2861 and Q2862). We did not include incidental appendectomy or the degree to which primary discharge diagnoses were confirmed by clinical, surgical, or pathologic findings.

### Definitions

The study population was defined as subscribers to the Korean National Health Insurance system from 2005 through 2007. Appendicitis comprised acute appendicitis (K35), other appendicitis (K36), and unspecified appendicitis (K37). Acute appendicitis (K35) is further classified as “with perforation, generalized peritonitis, or rupture (K35.0)”, “with peritoneal abscess (K35.1)”, and “without perforation, peritoneal abscess, peritonitis, or rupture (K35.9)”. Perforated appendicitis and perforated appendectomy were estimated using the procedure code because, in most cases, the procedure code is set immediately after surgery by the surgeon or physician and is therefore more accurate than the primary discharge diagnosis in indicating perforation of the appendix.

Total appendectomy refers to the sum of non-perforated appendectomies and perforated appendectomies. The total appendectomy rate was defined as the percentage of inpatients who underwent appendectomy among inpatients diagnosed with appendicitis, and the perforated appendectomy rate was defined as the percentage of perforated appendectomies among the number of appendectomies.

### Statistical analysis

Because the prevalence of appendectomy was not investigated, an estimate of the population undergoing appendectomy in each age group was calculated using the life table method to estimate the proportion of people in an age group who underwent an appendectomy for any reason before reaching the given age interval, assuming stability of age-specific appendectomy rates over the past 85 years.^7^

To compare the incidence of appendicitis in different months and seasons, months with fewer than 31 days were adjusted to fit a standard month of 31 days. To identify seasonal variation, the Fisher kappa (Fκ) and Bartlett Kolmogorov–Smirnov (BKS) tests were used to assess cyclic patterns in event occurrence on a monthly basis.^3^^,^^7^^,^^8^^,^^10^ The standardized lifetime risk developed by Sasieni et al was used to evaluate the secular trend in appendicitis and appendectomy.^11^ The Fisher exact test was used to compare the incidence between sexes.

Lifetime risk of appendicitis and appendectomy was defined as the number of population that will face appendicitis or undergo appendectomy by surviving to the age of 100 years. This was calculated by adjusting the risk for 10 000 people at a specific age with intact appendices, using the complete life table published by the Korean National Statistical Office.^2^^,^^12^ In computing the lifetime risk of appendicitis and appendectomy, the population who had already undergone appendectomy or who had died was excluded from the denominator.

*P* < 0.05 was regarded as statistically significant. All statistical analyses were performed using SAS version 9.1 (SAS Institute Inc., Cary, NC, USA).

## RESULTS

During the period from 2005 through 2007, among a study population of 142 621 326 people (approximately 47 540 000 per year), 310 961 (approximately 103 654 per year) received a diagnosis of appendicitis.

### Appendicitis

**Figure 1.**
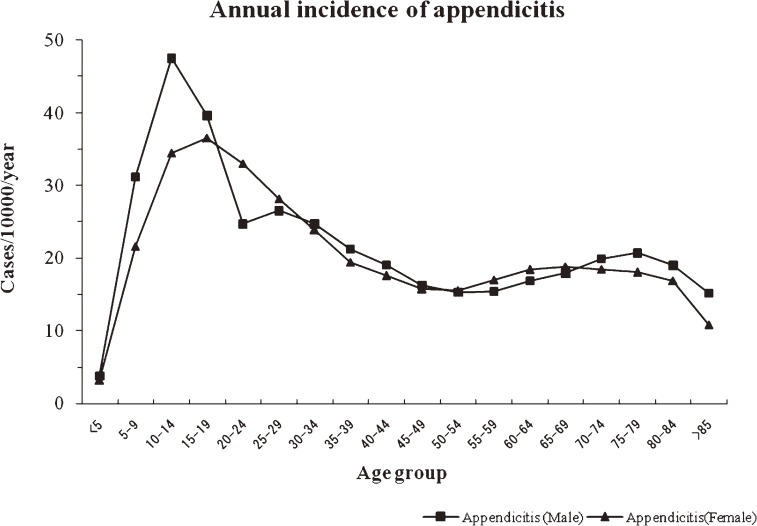
Annual incidence of appendicitis (per 10 000 population) in South Korea, by age group and sex, 2005–2007.

A total of 98.77% of patients who were diagnosed with appendicitis had acute appendicitis. The overall incidence of appendicitis was 22.71 per 10 000 population per year (95% CI: 22.42–23.01). The values were 23.58 per 10 000 population per year (95% CI: 23.28–23.88) for males and 21.81 per 10 000 population per year (95% CI: 21.53–22.10) for females (*P* > 0.05 between sexes). The age-specific incidence of appendicitis displayed a similar pattern in males and females (Figure 1).

The overall male-to-female ratio of the incidence of appendicitis was 1.08 (relative risk: 1.07, 95% CI: 0.60–1.91). The age range of highest incidence was 10–14 years (47.52 per 10 000 population per year, 95% CI: 47.10–47.94) for males and 15–19 years (36.55 per 10 000 population per year, 95% CI: 36.18–36.92) for females (*P* > 0.05 between sexes). The age range of lowest incidence in both sexes was those younger than 5 years (3.81 per 10 000 population per year, 95% CI: 3.69–3.93 in males; 3.18 per 10 000 population per year, 95% CI: 3.07–3.29 in females; *P* > 0.05 between sexes).

### Appendectomy

The overall incidence of total appendectomy was 13.56 per 10 000 population per year (95% CI: 13.33–13.78); the values were 14.28 per 10 000 population per year (95% CI: 14.05–14.51) for males and 12.81 per 10 000 population per year (95% CI: 12.59–13.04) for females (*P* > 0.05 between sexes).

**Figure 2.**
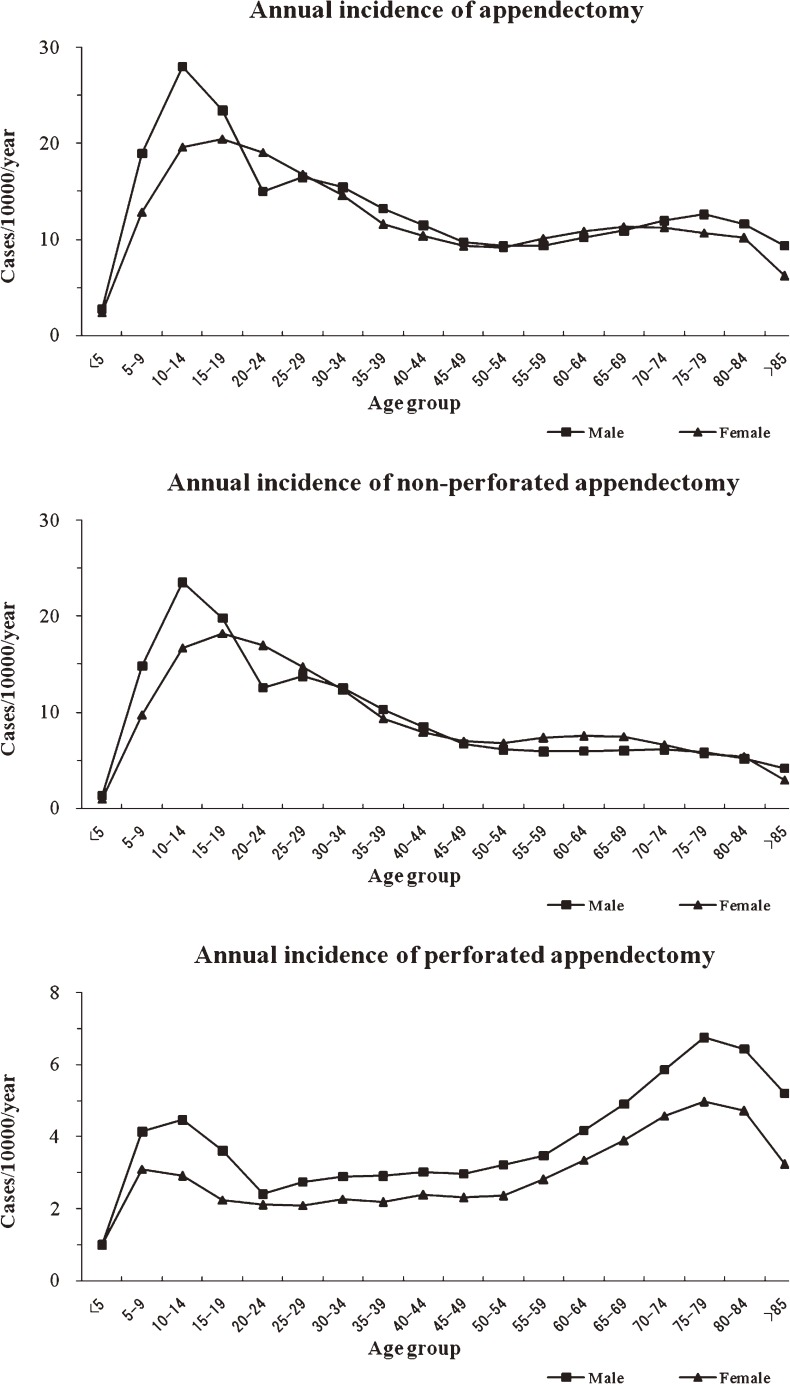
Annual incidence of total appendectomy, perforated appendectomy, and non-perforated appendectomy (per 10 000 population) in South Korea, by age group and sex, 2005–2007.

The age-specific incidence of total appendectomy displayed a similar pattern in males and females (Figure 2). The overall male-to-female ratio of total and non-perforated appendectomy was 1.11 (relative risk: 1.06, 95% CI: 0.50–2.25).

### Perforated appendectomy

The overall incidence of perforated appendectomy was 2.91 per 10 000 population per year (95% CI: 2.81–3.01); the values were 3.27 per 10 000 population per year (95% CI: 3.16–3.38) for males and 2.55 per 10 000 population per year (95% CI: 2.45–2.64) for females (*P* > 0.05 between sexes). The age-specific incidence of perforated appendectomy was similar between sexes (Figure 2).

The overall male-to-female ratio of perforated appendectomy was 1.29 (relative risk: 1.48, 95% CI: 0.25–2.83), and there was an M-shaped pattern for the age-specific incidence of perforated appendectomy. The second highest incidence was in the age range of 5–14 years, after which incidence declined to its nadir—between the ages of 20 and 24 years—and remained low until it began to increase again between the ages of 50 and 54 years. The highest incidence was in the range of 75–79 years of age, after which it declined again.

**Figure 3.**
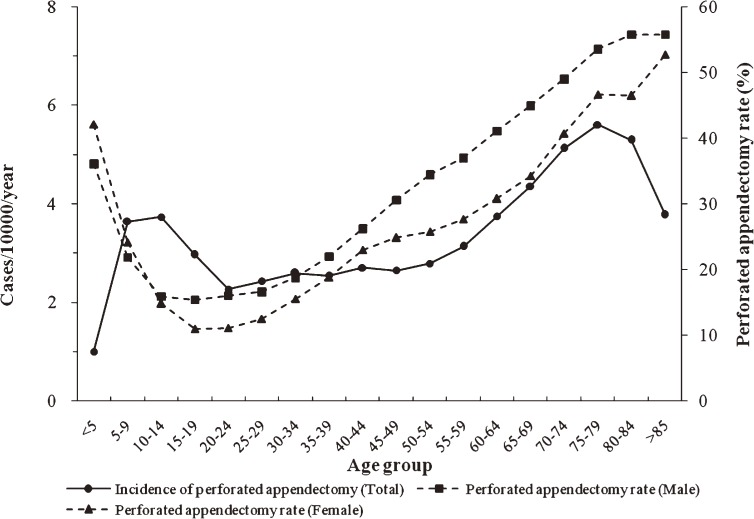
Overall incidence of perforated appendectomy and perforated appendectomy rate in South Korea, by age group and sex, 2005–2007.

The overall perforated appendectomy rate was 21.47% (95% CI: 21.19–21.76); the values were 22.90% (95% CI: 22.61–23.20) for males and 19.85% (95% CI: 19.58–20.13) for females (*P* > 0.05 between sexes). The age-specific perforated appendectomy rate was similar in males and females (Figure 3).

The perforated appendectomy rate was lowest in those aged 15–19 years; the values were 15.43% (95% CI: 15.19–15.67) for males and 10.98% (95% CI: 10.77–11.18) for females. As age increased, the rate also increased, finally reaching a peak at age older than 85 years (55.80%, 95% CI: 55.65–55.95 in males; 52.72%, 95% CI: 52.35–53.09 in females) in both males and females (*P* > 0.05 between sexes).

### Appendicitis and appendectomy

**Figure 4.**
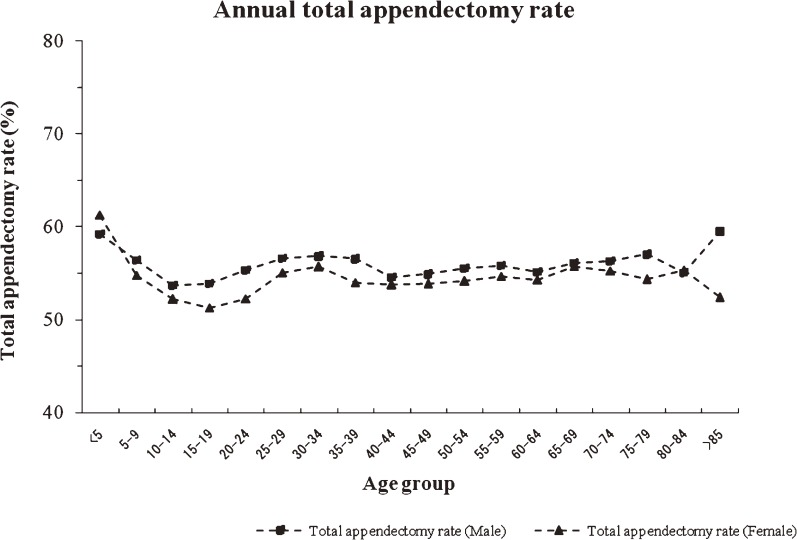
Annual total appendectomy rate in South Korea, by age group, 2005–2007.

Appendectomy was performed in 59.70% (95% CI: 59.22–60.18) of inpatients who received a diagnosis of appendicitis. The total appendectomy rate was 60.55% (95% CI: 60.07–61.03) for males and 58.76% (95% CI: 58.29–59.24) for females. Age-specific total appendectomy rates were similar in males and females (Figure 4).

The age-specific total appendectomy rate was highest at age younger than 5 years (60.99 per 10 000 population per year, 95% CI: 60.52–61.46 in males; 63.22 per 10 000 population per year, 95% CI: 62.75–63.70 in females) and the lowest rate was at age 15–19 years (59.13 per 10 000 population per year, 95% CI: 58.66–59.59 in males; 56.02 per 10 000 population per year, 95% CI: 55.56–56.47 in females). The rate increased slightly in those aged older than 19 years, but did not change after age 25–29 years. The rate did not differ between sexes (*P* > 0.05).

### Seasonal variation and secular trends, 2005–2007

**Figure 5.**
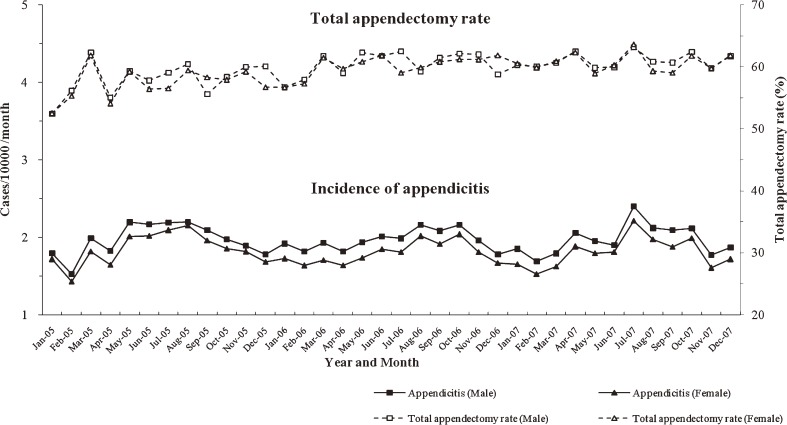
Incidence of appendicitis and total appendectomy rate in South Korea, by month and sex, 2005–2007.

**Figure 6.**
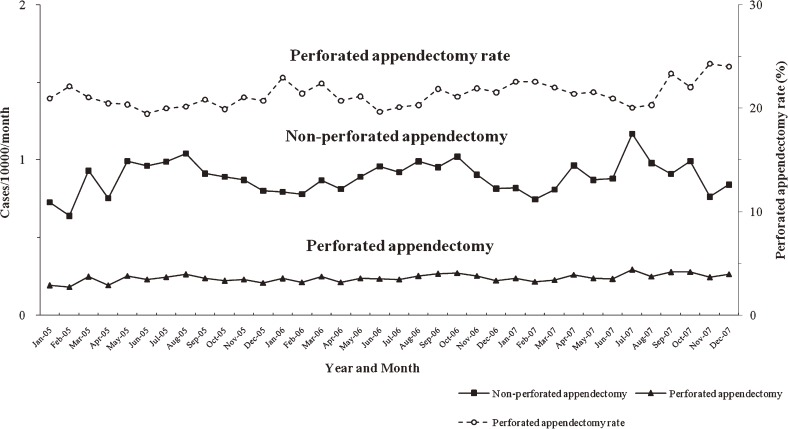
Incidence of non-perforated appendectomy, and perforated appendectomy (per 10 000 population), and perforated appendectomy rate in South Korea, by month, 2005–2007.

Analysis of the monthly incidence of appendicitis revealed clear seasonality in males (Fκ = 10.66; BKS = 0.52, *P* < 0.05) and females (Fκ = 11.11; BKS = 0.58, *P* < 0.05): peaks appeared during summer months and troughs during winter months (Figures 5 and 6). A similar pattern of seasonal variation was found for the incidence of total appendectomy (Fκ = 4.12; BKS = 0.46, *P* < 0.05) and non-perforated appendectomy (Fκ = 9.98; BKS = 0.48, *P* < 0.05). The perforated appendectomy ratio (Fκ = 7.20; BKS = 0.51, *P* < 0.05) also showed seasonality, although it peaked during winter, rather than summer, months. In contrast, the total appendectomy and perforated appendectomy rates did not show clear seasonality.

**Figure 7.**
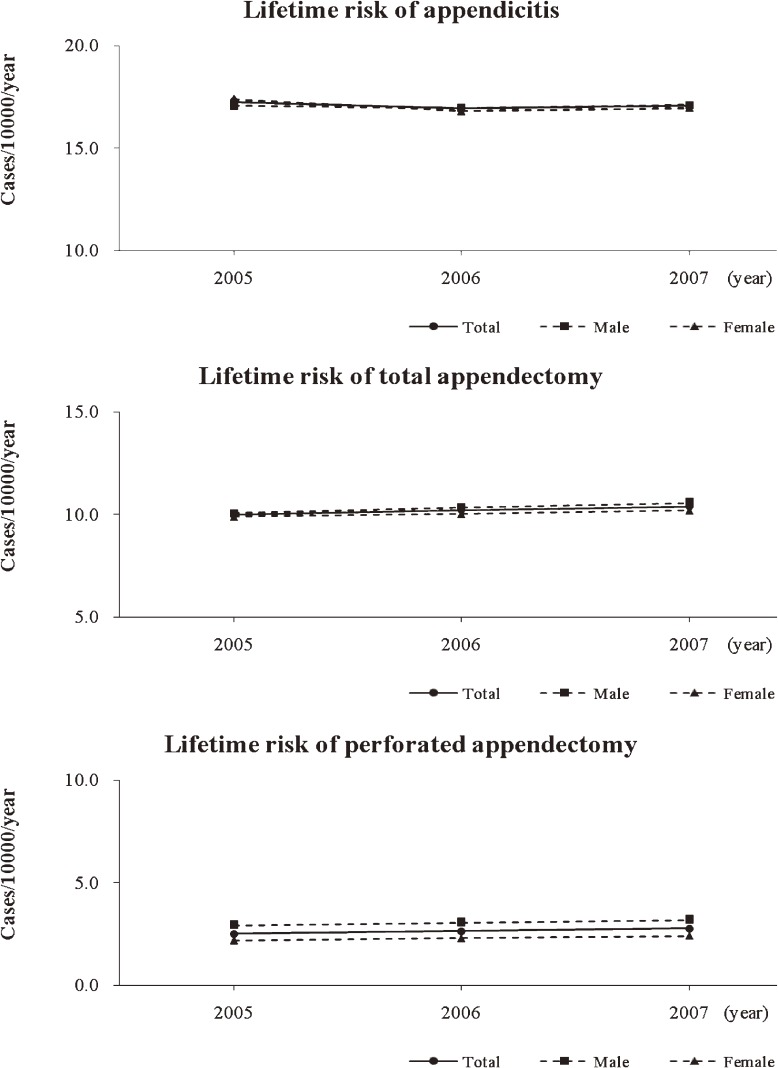
Standardized lifetime risk of appendicitis, total appendectomy, and perforated appendectomy.

The standardized lifetime risks for appendicitis, total appendectomy, and perforated appendectomy between 2005 and 2007 were analyzed to reveal the secular trends; these data are presented in Figure 7. The incidences of appendicitis, total appendectomy, and perforated appendectomy were constant during the reference period.

### Life table analysis: risk of appendicitis or appendectomy

The analysis assumed that males and females have a life expectancy identical to that recorded for 2006 and a constant incidence of appendicitis and appendectomy in 2005 through 2007. Among the 10 000 children younger than 5 years, the lifetime risks of appendicitis and appendectomy were 1632.88 and 989.15 individuals, respectively, for males, and 1634.44 and 961.20 individuals for females (Table 1). In other words, when incidental appendectomy is performed in 10 000 women aged 20 to 25 years, it will prevent 1148 patients with suspected appendicitis from being hospitalized and 683 patients from undergoing appendectomy.

**Table 1.** Cumulative lifetime risk (to age 100 years) for appendicitis and appendectomy per 10 000 population in South Korea

Age group (years)	Appendicitis		Total appendectomy	
	
	Males	Females	Males	Females
<5	1632.88	1634.44	989.15	961.20
5–9	1571.87	1591.70	950.69	934.39
10–14	1401.60	1470.82	849.02	864.37
15–19	1198.81	1309.49	729.77	773.61
20–24	1044.48	1147.70	638.08	682.35
25–29	927.83	1003.90	566.67	598.54
30–34	809.02	880.97	492.64	524.55
35–39	701.56	778.13	425.49	462.27
40–44	609.17	691.45	368.68	411.03
45–49	530.77	614.09	321.63	365.44
50–54	464.77	542.83	281.90	323.40
55–59	404.76	470.48	245.32	280.65
60–64	344.65	392.44	208.94	234.33
65–69	284.59	311.99	172.68	186.41
70–74	224.61	234.46	136.50	139.33
75–79	164.48	164.23	100.50	96.88
80–84	112.62	104.10	69.08	60.88
85–89	75.89	62.16	46.77	35.53
90–94	59.16	45.72	36.46	26.13
95–99	38.64	28.44	23.81	16.26

## DISCUSSION

In this study, the overall incidence of appendicitis was 22.71 per 10 000 population per year, which is higher than the previously reported values of 7.5 to 12.0 per 10 000 population per year.^2^^–^^4^^,^^6^^–^^8^ This may be because the present study and other studies used different definitions of appendicitis. In most previous studies, a diagnosis of appendicitis was restricted to patients who had undergone appendectomy; however, we applied a diagnosis of appendicitis to inpatients who had a discharge code of appendicitis, regardless of whether they underwent appendectomy. Therefore, strictly speaking, the overall incidence of appendicitis observed in our study refers to the incidence of inpatients with suspected appendicitis. The incidence of total appendectomy was estimated in this study using the same definition as that used in most other studies. The overall incidence of total appendectomy was 13.56 per 10 000 per year (range, 13.25–14.60), which is the upper limit of the rates reported in previous studies.^2^^–^^8^ This high incidence of appendicitis and appendectomy in South Korea was unexpected, given that Korean foods have a higher fiber content than foods common in the Western diet and that populations with a high fiber diet have lower reported incidences of appendicitis.^13^^–^^16^ The reason for this finding is unknown, but the following are possible explanations for the differences between the rates observed in this study and those reported previously. First, because all citizens are covered by national health insurance in South Korea, the burden of medical expenses on patients is relatively low, and health care workers have more freedom in deciding to perform an appendectomy for patients with suspected appendicitis. Thus, the diagnostic accuracy of appendectomy may be lower in South Korea. Second, the incidence of appendicitis in South Koreans may be higher than the rates reported previously, because of racial, geographic, and/or climatic differences.

As was the case in previous reports, the incidence of perforated appendectomy changed little with age. However, the second highest incidence was in those aged 5–14 years, after which it declined. Incidence then increased with age, and reached a peak in those aged 75–79 years (an M-shaped pattern; Figure 3). This result also differs from those of previous studies, which reported a peak incidence of perforated appendicitis in those aged 10–14 years, although Luckmann and Davis reported a similar M-shaped pattern for Asians living in California.^2^^,^^3^^,^^7^ Although the causes of these divergent findings are not clear, racial and ethnic factors are thought to contribute to the higher rate of perforated appendectomy in older individuals, as compared to teenagers.

As shown in Figures 5 and 6, the seasonal pattern in the incidence of appendicitis and appendectomy was similar to that noted in previous studies, which showed that incidence increased in summer.^1^^–^^3^^,^^7^^,^^17^ The rates of total appendectomy and perforated appendectomy did not show seasonality in our study: a constant proportion of inpatients who were hospitalized after receiving a diagnosis of appendicitis underwent appendectomy regardless of season. In addition, the perforated appendectomy rate was constant, despite the increased incidence of appendicitis and total appendectomy during summer. Several factors may contribute to the seasonality of appendicitis and appendectomy, but no single causative factor has been identified.^2^^,^^7^

Previous studies have reported a constant incidence of appendicitis and a decreasing incidence of appendectomy.^2^^,^^3^^,^^5^^,^^17^^,^^18^ In this study, the incidence of appendicitis and appendectomy were constant during the period from 2005 through 2007 (Figure 7). A similar secular trend was reported by Korner et al, who found a stable incidence of acute appendicitis in 2001.^19^ Factors that may influence the incidence of appendicitis and appendectomy are diagnostic accuracy, dietary change, socioeconomic status, hygienic standards, and access to health services.^2^^,^^5^ The incidence of appendicitis and appendectomy is thought to have decreased since the mid-20th century because of improvements in these factors.^2^^,^^3^^,^^5^^,^^17^^–^^19^ Although constant incidence of appendicitis and appendectomy were observed after the 1990s by Korner et al^19^ and in this study, the short observational period makes it difficult to draw a clear conclusion about the secular trend; further study is needed.

Because appendicitis can be prevented through incidental appendectomy, the preventive value for each age group, or the cost and benefit of incidental appendectomy, can be computed using the lifetime risk of appendicitis or appendectomy. Although some reports have computed the lifetime risk and preventive value of incidental appendectomy,^2^^,^^8^^,^^20^ this approach has several problems. For example, it is unclear whether the death rate caused by diseases other than appendicitis was included when calculating the preventive value of incidental appendectomy. Another problem is that previous studies did not include inpatients hospitalized with suspected appendicitis, because the targets were defined as inpatients who had undergone appendectomy. Inpatients with suspected appendicitis must be included when calculating the preventive value of incidental appendectomy, because the patients who underwent incidental appendectomy would not need to be hospitalized for suspected appendicitis. We considered these potential problems when calculating the lifetime risk of appendicitis and appendectomy, and we adjusted the data accordingly using the life table. We calculated the lifetime risk of inpatients hospitalized for suspected appendicitis and those who received an appendectomy. We believe that the lifetime risk computed in this study more accurately reflects the preventive value of incidental appendectomy. A separate study on this matter is in progress and will be reported in future.

We analyzed longitudinal, population-based, and national registry data to explore the epidemiological features of appendicitis in South Korea. The strength of our study is that it was conducted for 3 years and included almost the entire population of South Korea as the study population. However, our study has several important limitations. First, the HIRA data set may include some misclassification or coding errors for the discharge diagnosis and the surgical procedure, as was the case for several prior studies using national data.^2^^,^^3^^,^^5^^,^^7^^,^^9^ Because the health insurance system of South Korea does not acknowledge incidental appendectomy, incidental appendectomies performed together with other major procedures may have been miscoded as non-incidental appendectomy. In other words, a portion of the incidental appendectomies may have been reported as appendectomy for appendicitis, either intentionally or unintentionally, and a portion of asymptomatic patients with histopathologically confirmed appendicitis after incidental appendectomy may have been reported as having undergone appendectomy for appendicitis. Second, to calculate prevalence of appendectomy in our study, we assumed that the age-specific appendectomy rate had been stable over the past 85 years. Because previous studies show a secular decrease in appendicitis and appendectomy, our assumption could have resulted in significant underestimation of the prevalence of appendectomy. Consequently, the possibility of underestimation in the incidence of appendicitis and appendectomy is probable.

In conclusion, as compared to studies of Western populations, our study of the South Korean population revealed similar results regarding incidence: people aged 10 to 19 years had the highest incidence of appendicitis and appendectomy, incidence was higher in males, and perforation rate and seasonality were both comparable. However, the overall incidence of appendicitis and appendectomy was slightly higher among South Koreans. From 2005 through 2007, the incidence of appendicitis and appendectomy was constant. Overall, an estimated 15 incidental appendectomies are performed to prevent 1 inpatient with suspected appendicitis, and 26 incidental appendectomies are performed to prevent 1 appendectomy. Thus, incidental appendectomy may have greater preventive value in Koreans.

## References

[1] Noudeh YJ , Sadigh N , Ahmadnia AY Epidemiologic features, seasonal variations and false positive rate of acute appendicitis in Shahr-e-Rey, Tehran . Int J Surg. 2007;5:95–8 10.1016/j.ijsu.2006.03.009 17448972

[2] Addiss DG , Shaffer N , Fowler BS , Tauxe RV The epidemiology of appendicitis and appendectomy in the United States . Am J Epidemiol. 1990;132:910–25 2239906 10.1093/oxfordjournals.aje.a115734

[3] Al-Omran M , Mamdani M , McLeod RS Epidemiologic features of acute appendicitis in Ontario, Canada . Can J Surg. 2003;46:263–8 12930102 PMC3211626

[4] Andersson R , Hugander A , Thulin A , Nyström PO , Olaison G Indications for operation in suspected appendicitis and incidence of perforation . BMJ. 1994;308:107–10 8298378 10.1136/bmj.308.6921.107 PMC2539237

[5] Blomqvist P , Ljung H , Nyrén O , Ekbom A Appendectomy in Sweden 1989–1993 assessed by the Inpatient Registry . J Clin Epidemiol. 1998;51:859–65 10.1016/S0895-4356(98)00065-1 9762879

[6] Körner H , Söndenaa K , Söreide JA , Andersen E , Nysted A , Lende TH , Incidence of acute nonperforated and perforated appendicitis: age-specific and sex-specific analysis . World J Surg. 1997;21:313–7 10.1007/s002689900235 9015177

[7] Luckmann R , Davis P The epidemiology of acute appendicitis in California: racial, gender, and seasonal variation . Epidemiology. 1991;2:323–30 1742380 10.1097/00001648-199109000-00003

[8] Sugimoto T , Edwards D Incidence and costs of incidental appendectomy as a preventive measure . Am J Public Health. 1987;77:471–5 10.2105/AJPH.77.4.471 3826467 PMC1646952

[9] Kim HA , Kim S , Seo YI , Choi HJ , Seong SC , Song YW , The epidemiology of total knee replacement in South Korea: national registry data . Rheumatology (Oxford). 2008;47:88–91 10.1093/rheumatology/kem308 18077497

[10] Fuller WA. Introduction to statistical time series. New York: John Wilcy; 1976.

[11] Sasieni PD , Adams J Standardized lifetime risk . Am J Epidemiol. 1999;149:869–75 10221324 10.1093/oxfordjournals.aje.a009903

[12] Kleinbaum DG, Kupper LL, Morgenstern H. Epidemiologic research: principles and quantitative methods. New York: NY; Van Nostrand Reinhold Company; 1982.

[13] Adamidis D , Roma-Giannikou E , Karamolegou K , Tselalidou E , Constantopoulos A Fiber intake and childhood appendicitis . Int J Food Sci Nutr. 2000;51:153–7 10.1080/09637480050029647 10945110

[14] Black J Acute appendicitis in Japanese soldiers in Burma: support for the “fibre” theory . Gut. 2002;51:297 10.1136/gut.51.2.297 12117902 PMC1773321

[15] Brender JD , Weiss NS , Koepsell TD , Marcuse EK Fiber intake and childhood appendicitis . Am J Public Health. 1985;75:399–400 10.2105/AJPH.75.4.399 2983577 PMC1646243

[16] Burkitt DP , Moolgaokar AS , Tovey FI Aetiology of appendicitis . Br Med J. 1979;1:620 10.1136/bmj.1.6163.620 427491 PMC1598350

[17] Primatesta P , Goldacre MJ Appendicectomy for acute appendicitis and for other conditions: an epidemiological study . Int J Epidemiol. 1994;23:155–60 10.1093/ije/23.1.155 8194912

[18] Williams NM , Jackson D , Everson NW , Johnstone JM Is the incidence of acute appendicitis really falling? Ann R Coll Surg Engl. 1998;80:122–4 9623378 PMC2502999

[19] Körner H , Söreide JA , Pedersen EJ , Bru T , Söndenaa K , Vatten L Stability in incidence of acute appendicitis. A population-based longitudinal study . Dig Surg. 2001;18:61–6 11244262 10.1159/000050099

[20] Wang HT , Sax HC Incidental appendectomy in the era of managed care and laparoscopy . J Am Coll Surg. 2001;192:182–8 10.1016/S1072-7515(00)00788-2 11220718

